# Solar neighborhoods: the impact of urban layout on a large-scale solar strategies application

**DOI:** 10.1038/s41598-023-43348-8

**Published:** 2023-11-01

**Authors:** Kuljeet Singh, Caroline Hachem-Vermette, Ricardo D’Almeida

**Affiliations:** 1 Future Urban Energy Lab for Sustainability (FUEL-S), Faculty of Sustainable Design Engineering (FSDE), University of Prince Edward Island, 550 University Ave, Charlottetown, PE C1A 4P3 Canada; 2 Solar Energy and Community Design Lab, School of Architecture, Planning and Landscape (SAPL), University of Calgary, 2500 University Drive NW, Calgary, AB T2N 1N4 Canada; 3 Department of Building, Civil and Environmental Engineering, Gina Cody School of Engineering and Computer Science, Concordia University, 1455 de Maisonneuve Blvd West, Montreal, QC H3G 1M8 Canada

**Keywords:** Solar energy, Energy infrastructure

## Abstract

The article addresses the challenges of evaluating energy performance in different neighborhood settings under various energy efficiency measures and proposes a methodology for selecting appropriate solar strategies on a neighborhood scale. The study selects five representative neighborhoods from various climatic zones with different building and street layouts. The proposed methodology involves a systematic three-step multi-domain workflow for implementing energy efficiency measures and solar strategies in the existing neighborhoods. The first step involves typical energy performance simulation, the second step involves energy simulation using high performance building envelope, and the third step involves the addition of solar strategies in combination with retrofitting materials to achieve net-zero status. The results of the study show that modifying the building envelope leads to a significant reduction in energy consumption, with up to 60% reduction observed. The study also finds that the optimal mix of solar strategies depends strongly on the type of neighborhood, its street layouts, and the type of buildings. The article highlights the importance of considering these factors when implementing solar strategies on a neighborhood scale to achieve energy efficiency and net-zero status. It provides urban planners with a systematic decision-making approach to evaluate and optimize neighborhoods to achieve net-zero energy status.

## Introduction

The operational energy demand of buildings is responsible for 30% of the energy use worldwide^1^. Energy consumption and solar energy generation capacity in urban settings are key components that need to be well integrated into the design of buildings and neighborhoods, both new and existing, to achieve significant energy and GHG emission reduction goals^2^.

Photovoltaics (PV) application in buildings has been vastly researched, worldwide^3, 4^. D’Adamo et al.^5^ evaluated that PV has low risk source of solar energy with high economic returns. It is evident that there is an essential need to implement more sustainable ways of generating energy due to the expected shortage of fossil fuels in the future. Moreover, renewable energy has an infinite power supply that is less expensive and has a lesser environmental effect^6^. Renewable energy sources are expected to produce between 49 and 67% of primary energy by 2050^7^. Achieving net zero energy in urban districts and neighborhoods require the prominent adoption of renewable energy installation on the urban scale. For instance, various neighborhood surfaces can be used to install solar technologies. Utilizing solar energy to generate electricity have numerous advantages, including financial independence from utility companies, government incentives, the potential to profit from excess energy production by selling power back to utility companies, and less environmental effect^8^. In this context, profitability is governed by prosumers-based market and their self consumption drives the future energy transitions in cities^9^. Further, employing photovoltaics to generate electricity is critical in supplying distributed renewable energy to large urban centers, using building surfaces that receive direct solar radiation^10^. On a larger scale urban environment, it points to great financial savings, having a payback estimated in nearly 7 years or less, depending on the performance of the systems^11^.

Recently, the term “solar neighborhoods” attracted researchers’ interest and it refers to urban developments that use passive solar methods, as well as solar energy technologies (photovoltaic and thermal collectors) to reduce energy consumption as well as to generate enough power to meet their energy requirements. Solar neighborhood designs must consider critical variables such as building forms, buildings’ density, and site layout in order to maximize solar potential^12^. Renewable energy sources, particularly solar energy, are becoming increasingly important in the design of new energy-efficient buildings aiming for net zero energy status^13^ and energy resilience^14^. On a neighborhood scale, other aspects beyond the energy performance of individual buildings must be addressed, such as mutual shading of buildings, urban density, public illumination, etc.^15^. Thus, the street layout affects building shape and building orientation, and as such it plays a significant part in solar energy harvesting, leading also to an impact on building energy and environmental performance^16^. Depending on the layout and design, a neighborhood may save up to 65 to 85% of its energy demand after implementing solar systems^12^. For instance, the spinal cord of a neighborhood is its street layout, as it influences the flow of traffic, building orientation, solar surfaces, passive design, and interconnectivity^17^, and thus has a direct impact on energy efficiency. Furthermore, the street layout has a significant impact on the amount of energy consumption and GHG emissions associated with transportation, which combined with buildings, are the major global energy consumers^18^.

Literature shows multiple ways of evaluating energy performances at a neighborhood scale and it all depends on the focus of each research. For instance, to develop energy models that simulate a building or plant model for predicting loads or cost savings, a number of strategies are being created^19^. Barbosa et al.^20^ proposed a methodology to evaluate the cost-effectiveness of building retrofitting influenced by embodied energy and carbon emissions. Walker et al.^21^ presented a scenario-based method to evaluate the performance of solar PV and heat pump installation on the neighborhood scale. Moreover, energy performance can be interpreted in various ways, like buildings’ energy demand, solar potential, environmental impact, and pollutants emission. Marique and Reiter^22^ proposes a simplified approach and considers just the energy demands of buildings, transportation, and public lighting. Energy performance evaluation needs to emphasize the integration of energy action planning and urban design^23^. Another methodology weighs on the impact that urban design has on transportation and its greenhouse gasses (GHG) emissions^17^. Several researchs analyzed the solar access of urban areas and neighborhood designs, in different countries around the world employing various simulation tools, such as Energy Plus, e-QUEST, Simergy, and others. Ascione et al.^24^ proposed an EnergyPlus-based methodology to evaluate the impact of various energy efficiency measures such as retrofitting, heating ventilation, and air-conditioning, photovoltaics. To develop neighborhood models CAD Mapper and SketchUp are used. However, the study is mainly focused on the neighborhood with apartment buildings only. Asif et al.^11^ used ArcGIS and PVSyst to evaluate the performance of roof-mounted photovoltaic (PV) panels on university campus buildings. A study presented by^25^ used Daysim and CitySim to estimate the potential of building integrated photovoltaics (BIPV) with the level of detail (LoD) 2 model.^26^ proposed a methodology to evaluate the neighborhood’s social impacts upon adopting solar panels.^27^ studied the impact of street layout on the solar access and resilience of the representative hypothetical neighborhoods.

In the existing literature, for solar energy adoption on the neighborhood scale limited types of buildings are considered (e.g., the neighborhood with apartment buildings only). Also, the impact of energy efficiency measures and solar strategies is considered simultaneously in very few papers. For instance, in most works of literature, only rooftop solar energy systems are considered for a given type of building such as campus buildings or apartment buildings. Literature available for neighborhood energy modeling proposes simplified methodologies due to problems of extracting building features at the neighborhood scale, hence limited literature is available with level of detail (LoD) 3 neighborhood-level simulations. Therefore, a structured matrix for data collection is needed that will help energy modelers to setup neighborhood-scale energy models. Also recently, a review article by^28^ presented studies on solar energy accessibility in built environment at high latitude locations (such as Canada). The article reported that urban building envelope-based numerical studies to identify solar exploitation is the most researched topic ignoring outdoor and indoor urban spatial domains, since there is lack of multi-domain studies in the literature. The street layout can impact the energy and solar performance of the neighborhoods as well^27^. The impact of street layouts on performance and solar energy harvesting is not clearly correlated in the existing literature as well. In addition, a decision-making framework for selecting appropriate solar strategies on the neighborhood scale is not well explored in the literature.

This paper aims at evaluating the impact of energy efficiency measures as well as various solar strategies selection that maximize onsite energy generation, in various neighborhood archetypes with diverse types of buildings (detached houses, duplexes, townhouses, and multi-family apartment buildings), representatives of North American neighborhoods. It presents a systematic methodology to evaluate existing neighborhoods using LoD 3 models as well as to estimate the impacts of different urban layouts on the energy performance and solar generation potential in an urban environment. The paper also presents a novel approach for urban planners and energy modelers to extract data from existing neighborhoods including geometric information, that is employed to analyze energy efficiency measures, and quantify the effect of implementing solar strategies/energy. Addressing the lack of multiple urban spatial domains studies in the existing literature^28^, the proposed workflow considers various urban spatial domains simultaneously such as envelope (evaluating solar access and active energy potential), outdoor (to identify outdoor surfaces for solar installations), and indoor (impact of weather and solar access on indoor energy consumption upon retrofitting). The paper also presents a simple decision-making technique that assists in determining the best strategies for specific neighborhoods. The work can give an insight into the impact of urban planning on neighborhoods, on their energy performance and on their capacity to integrate solar strategies to achieve a net zero energy status, and how to make decisions on the application and suitability of these strategies. In addition to application of this study for net-zero energy neighborhoods, it can be also useful in envisioning energy positive developments as solar energy is identified as a key pillar in the literature^29^.

## Material and methods

This section describes the methodology of the paper including neighborhood selection, energy modeling, simulation details, and solar strategies. Firstly, a selection of five neighborhoods representative of common urban layouts in Canada (and North America) are analyzed. The assessed neighborhoods are all residential. The archetypes that compose each neighborhood are detached houses, attached houses (duplexes and townhouses), and multi-family apartment buildings. A 3D model of each of these neighborhoods is developed to assess the solar potential and have a base to develop an energy model. Energy Plus in conjunction with SketchUp (using a plugin-Euclid) is employed in development.

**Figure 1:**
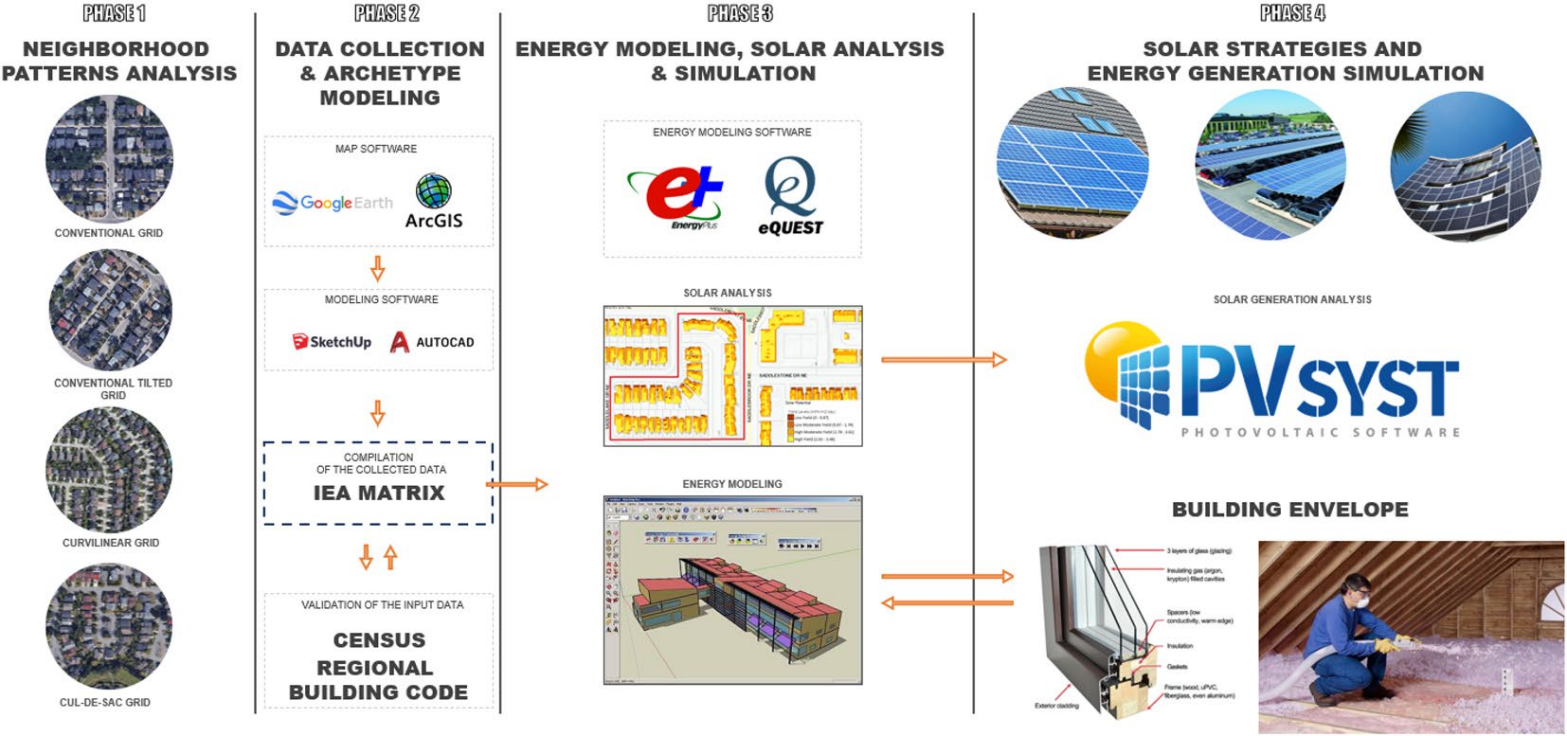
Overall workflow of proposed methodology.

To develop an energy model, a baseline is developed using the Natural Resources Canada energy use database for validation purposes^30^, which has the average energy use based on building types, building use, and heating and cooling systems, divided by each province. Energy simulations are performed to estimate the demand of each of the selected neighborhoods and validated against the baselines. These models are then systematically modified to reduce the overall energy consumption and to implement various solar strategies. The study focuses on the reduction of heating loads by altering the materials of the building envelope. Energy consumption of the neighborhoods is compared to energy generation to determine whether a net-zero energy status can be achieved. Figure 1 illustrates the methodology workflow.

A matrix is used to classify, evaluate, and understand the neighborhood characteristics in a study developed by the International Energy Agency (IEA) Task 63: Solar Neighborhood Planning (International Energy Agency, 2022). This matrix exhibits five different types of street layouts that represent North American street networks: conventional grid, conventional grid with tilted orientation, curvilinear loop, cul-de-sac, and radial. Street layouts have an immediate impact on the solar generation potential of a neighborhood since they affect the design and set of buildings.

### Matrix for neighborhood characterization

Cities are composed of districts, which all have distinct characteristics, but most of them morph into urban patterns that can be visually identified. The International Energy Agency (IEA) Task 63: Solar Neighborhood Planning developed a matrix to catalog different patterns of neighborhoods, classifying them by neighborhood types and uses, street layouts, building designs, and several other neighborhood parameters to recognize such patterns. The matrix is based on an extensive spreadsheet that allows inputting data related to the features of neighborhoods, separated into six categories:

A) *Neighborhood type*: this section displays general data including total area, demographics such as population, number of dwellings, as well as street layout characteristics, type of neighborhood (e.g., residential, mixed-use), and green areas.
B) *Neighborhood building structure and passive design*: related to the details of building block design as well as individual building designs, number of stories, units, and area. This section tackles as well as roof details, construction materials, the orientation of the façades, and window design.
C) *Solar energy generation*: this part includes various parameters that affect of the design of solar technologies (photovoltaic and thermal collector systems), like orientation, tilt angle, efficiency, and surface location.
D) *Energy systems*: this section displays the energy systems employed in the buildings of the analyzed neighborhood, divided by heating, cooling, and water heating systems.
E) *Other information*: this section shows other features that might be relevant to the energy model, such as lighting, systems set points, and appliances.
F) *Simulation outputs*: this part presents a summary of the results simulated in the energy model.

Overall, using the IEA matrix as a template, this study aims to understand how the different aspects of a neighborhood impact energy performance, especially in the application of solar strategies (a detailed matrix is provided in Appendix A).

### Neighborhood selection

An analysis of urban patterns is performed to identify representative layouts across multiple cities in Canada. Various uncontrollable parameters such as climate, topography, and usage patterns are similar in all the selected neighborhoods. Based on this extensive analysis using satellite images, five different street layouts are assumed as representative of urban planning in Canada, as shown in Table 1, below. This study includes only existing residential neighborhoods of single-family residential units including different types of units. Residential units are divided into three categories such as single detached houses, single attached houses, and apartment buildings.

**Table 1:** Representative street layouts for selected neighborhoods^31^.

Conventional grid	Conv. grid–tilted orientation	Curvilinear loop	Cul-de-sac conventional	Radial
				

Five neighborhoods in different provinces are selected as a case study for each layout representative of urban patterns in Canada. The neighborhoods analyzed are Brighouse located in Richmond-BC, Parkdale, located in Calgary-Alberta (AB), Glendale, located in Winnipeg-Manitoba (MB), East York, located in Toronto-Ontario (ON), and Mount Royal, located in Montreal-Quebec (QC). Figure 1 shows the selected area of each neighborhood.

A small sample of each neighborhood is analyzed to ease the simulation process, ranging from 1.7 to 4.8 hectares (17,000–48,000 m^2^) due to the form of the block, which is affected by the street layout and type of construction. Density and demographic indicators, as well as other data from each community profile, are extracted from the Canada Census 2016^32^ and Natural Resources Canada 2019 database^30^. Characteristics of the selected neighborhoods are presented in Table 2.

**Table 2:** Selected neighborhood's characteristics.

Neighborhood	Location	Coordinates	Elevation (m)	Climate zone	Street Layout	Dwellings	Detached/attached house Ratio (%)	Avg floor area	Neighborhood area [ha]	Avg population per dwelling
Brighouse	Richmond, BC	49.165166°, -123.152489°	9	8b	Curv. Loop	44	100/0	229	3.32	2.7
Parkdale	Calgary, AB	51.058873°, -114.132373°	1059	4a	Conv. Grid – Tilted	25	68/32	138	1.69	2.2
Glendale	Winnipeg, MB	49.871385°, -97.328797°	239	3a	Cul-de-sac	24	100/0	291	3.23	2
East York	Toronto, ON	43.689285°, -79.337505°	121	6a	Conv. Grid	44	100/0	115	2.13	2.36
Mount Royal	Montreal, QC	45.517257°, -73.653455°	220	5a	Radial	40	90/10	347	4.8	2.3

### Energy modeling

**Figure 2:**
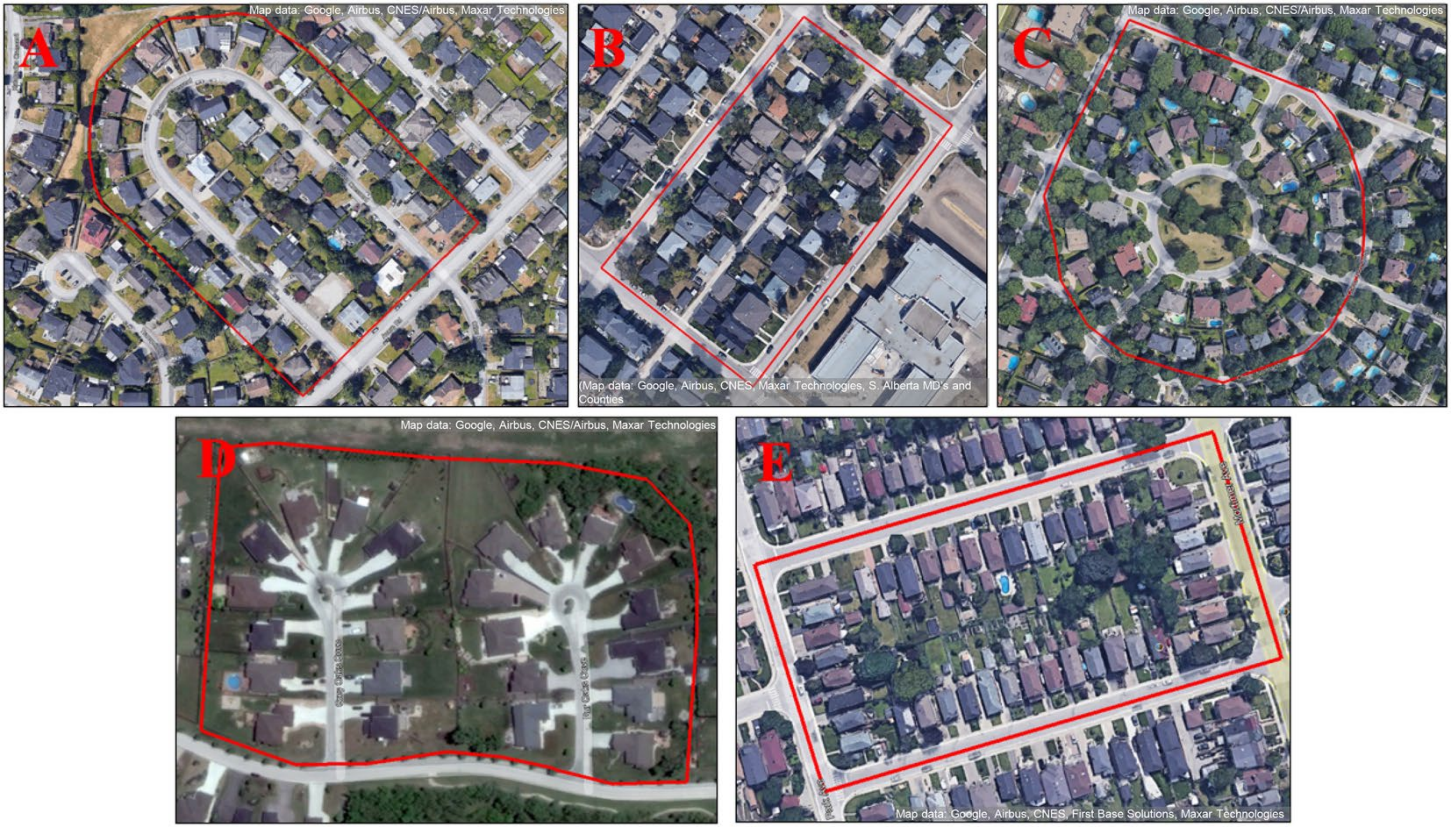
Aerial pictures of selected neighborhoods (taken from Google Earth): (**A**) Brighouse, (**B**) Parkdale (**C**) Mount Royal Brighouse, (**D**) Glendale, and (**E**) East York.

This section presents the assumptions employed in the energy modeling process of the selected neighborhoods, such as materials used, the development of a comparable baseline, and some rules followed to develop the 3D model in the energy simulation (Fig. 2).

#### Energy modeling assumptions

**Figure 3:**
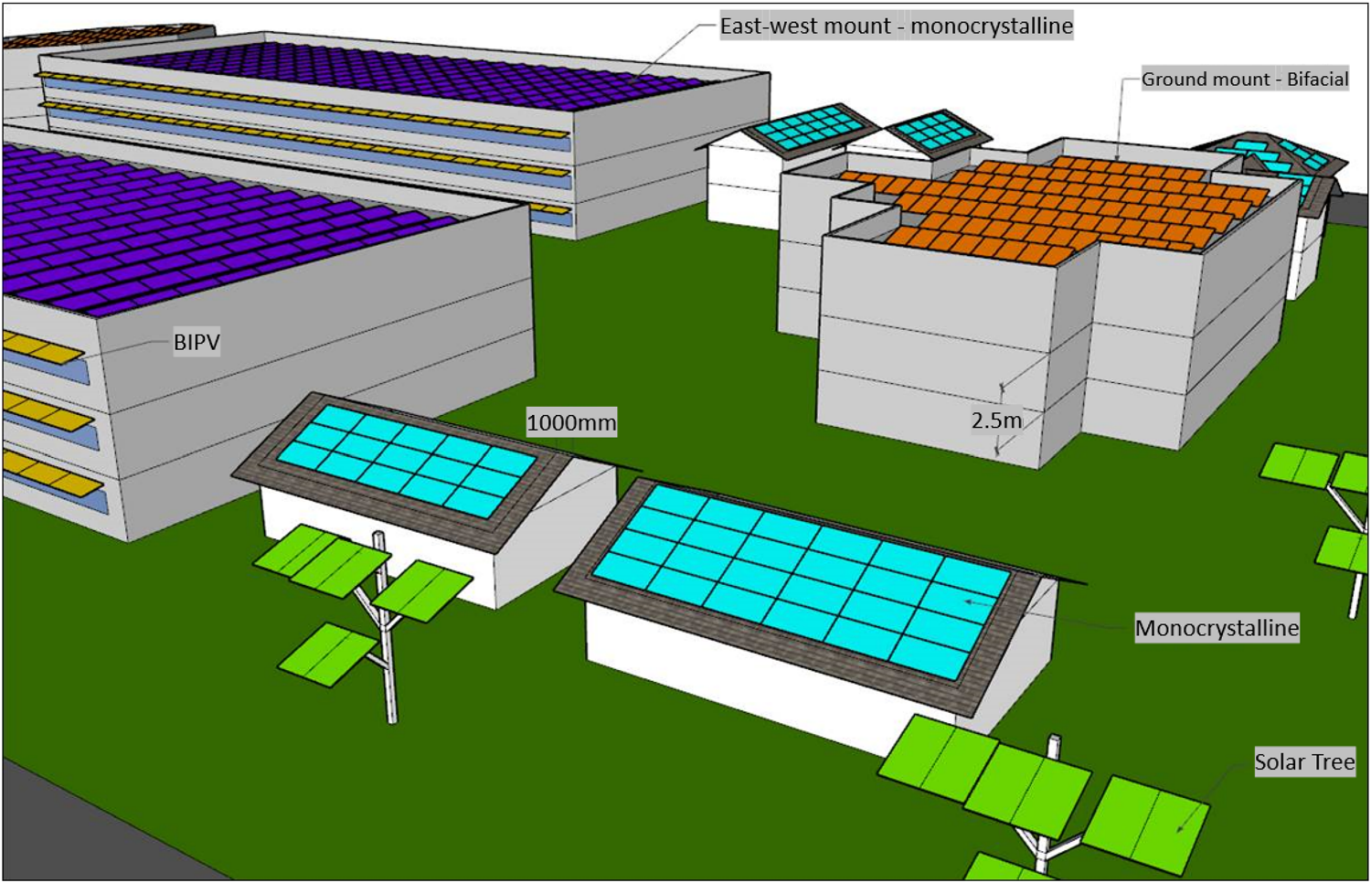
Visual representation of various solar strategies modeling assumptions.

The energy modeling process consists of first inputting all the obtained data into an energy modeling program to generate a comprehensive energy model of the neighborhood. EnergyPlus^33^, an energy analysis and thermal load simulation program, is utilized for this investigation. SketchUp and the plugin Legacy Open Studio^34^ are also used in conjunction with EnergyPlus to simplify the input of geometric data of all the buildings within a neighborhood. Euclid plugin uses SketchUp's modeling platform to transform geometric data (created in SketchUp) data from a 3D model into an EnergyPlus-compatible energy model. Satellite imagery is used to compute measurements for 3D modeling of neighborhood buildings. Furthermore, Google Street View function and 3D models from the Google Earth^35^ are used to represent the structures' rooftops as exactly as possible. The neighborhood energy models are formulated for this study are simulated as whole building stock to account the mutual effects of buildings. In the energy simulations, hourly weather data provided by Canadian energy and engineering data sets (CEEDS) is used for this work. In the development of the neighborhood models, the following assumptions were considered and can be also correlated from Fig. 3.

- Intending to ease the neighborhood modeling, the roof shape determines each building envelope forms, assuming external walls are 1 m offset from the roof edge.
- Each storey is assumed to have a ceiling height of 2.5 m, 0.40 m higher than the minimum required by the Ontario Building Code for room ceiling heights^36^. Building interior walls and internal divisions were not considered.
- If there are no street views available from Google Earth, fenestrations are based on the patterns of surrounding buildings.
- Each dwelling in a single-family structure is a zone, whereas multi-family buildings include a zone for each unit as well as a common space.
- Due to its widespread use in North American homes^30^, the heating system used in all energy simulations considered is the natural gas furnace with 80% efficiency considering ideal loads in order to standardize the simulation process. Heating setpoint is taken as 20 °C for all buildings.
- Because cooling systems are less commonly used in Canada in residential buildings due cold climates, space cooling systems are not taken into consideration.
- The building envelope is the sole upgrade researched and implemented for buildings retrofit.

Further, a single neighborhood building energy model (for each of the considered neighborhood) is constructed with different buildings and simulated in this work. It is essential to capture the mutual impact of buildings as well as the effect of the configuration of the neighborhoods on energy simulations.

#### Building envelope materials

To estimate the energy demand of the neighborhoods, two sets of simulations are proposed for the study. The first simulation set, referred to as “typical simulation,” uses standard materials applied in the North American construction sector. Those materials consist of insulated wood frame walls, plywood floors, asphalt shingle roofs coated with oriented strand board (OSB) boards, and double pane glazing. The second simulation set is conducted based on high-performance materials that present a superior R-value, referred as “high-performance simulation. The fenestration materials use the typical fenestration characteristics chart available in the American Society of Heating, Refrigerating and Air-Conditioning Engineers (ASHRAE) Handbook Fundamentals as a reference^37^. For the energy simulations, two glass layers with operable aluminum frames are used for the typical simulation, while triple-layered low-e, low-solar glazing with operable aluminum frames is assumed for high-performance simulation. This study considers only the heating load reduction when proposing the implementation of new building envelope materials. Table 3 illustrates the characteristics of the materials assumed in the simulations.

**Table 3:** Opaque building materials comparison.

Opaque materials				
	Name	Thickness [m]	Conductivity [W/m–K]	R-Value [m^2^-K/W]
TYPICAL: Wall	Synthetic stucco	0.003	0.865	0.040
	Sheathing consol layer	0.013	0.094	0.140
	7/16″ OSB	0.011	0.116	0.100
	Wall consol layer	0.140	0.057	2.440
	1/2″ drywall	0.013	0.160	0.080
	Total	0.180	0.514	2.80
HP: Wall	Synthetic stucco	0.003	0.865	0.040
	Sheathing consol layer	0.013	0.094	0.140
	7/16″ OSB	0.011	0.116	0.100
	HP wall insulation	0.330	0.050	6.730
	1/2″ drywall	0.013	0.160	0.080
	Total	0.370	1.285	7.090
TYPICAL: Roof	Asphalt shingle	0.010	0.082	0.077
	1/2″ OSB board	0.010	0.116	0.109
	Total	0.020	0.198	0.186
HP: Roof	Roof membrane	0.010	0.160	0.060
	HP roof insulation	0.690	0.050	14.040
	Total	0.700	0.210	14.100

#### Model validation

Following the neighborhood simulations, the database created by Natural Resources Canada for energy use statistics^30^ is employed to develop baselines to compare and validate the simulations. The comparison is conducted based on energy consumption per area and end-use (kWh/m^2^/year).

The database stated above has a great deal of information regarding energy use, broken down by province and building type. Three baseline models are built for each province for this study, based on the following building types: single detached houses, single attached houses, and apartment buildings Space heating, water heating, appliances, and lighting are among the end-uses evaluated. Whilst all these end-uses contribute to the overall energy demand of buildings; this research focuses on measures to minimize space heating demand since it is the most significant in Canada and is directly tied to building envelope design. By combining data from the database with the Canada Census 2016^32^, it is possible to estimate the energy demand per area for each baseline model. Table 4, based on the NRCan database set^30^ shows the assumptions for each baseline model.

**Table 4:** NRCan database—end-use energy consumption ^30^.

kWh/m^2^/year	Space heating	Water heating	Appliances	Lighting	Space cooling
British Columbia					
Single detached	91.71	28.95	22.93	8.52	1.47
Single attached	65.27	36.43	28.08	5.31	1.52
Apartments	45.37	43.78	34.62	4.38	0.80
Alberta					
Single detached	197.31	50.57	25.28	8.71	0.51
Single attached	98.51	44.37	23.07	5.32	0.00
Apartments	117.41	47.25	25.77	2.15	0.00
Manitoba					
Single detached	148.95	39.14	38.05	9.78	11.42
Single attached	115.74	28.94	34.72	5.79	0.00
Apartments	87.06	35.24	39.39	4.15	6.22
Ontario					
Single detached	144.23	36.00	19.39	6.71	10.49
Single attached	117.49	39.09	22.27	4.80	6.55
Apartments	93.81	47.08	27.45	2.08	3.30
Quebec					
Single detached	178.41	12.59	33.01	10.60	4.97
Single attached	117.24	31.31	35.97	9.33	4.66
Apartments	101.41	27.06	37.17	4.67	1.40

### Solar strategies application

Solar strategies applications are simulated employing PVsyst. Prior to running these simulations, a shading analysis is carried out employing SketchUp in order to ensure that solar systems are not shaded, for at least 7 h per day during the whole year. This analysis is crucial to understand the surroundings before designing solar strategies for each neighborhood, to optimize the energy generation of the photovoltaic modules. The shading analysis is used to recognize some of the environmental elements, like trees, other buildings, or different parts of the roof, that may shade the PV system.

**Figure 4:**
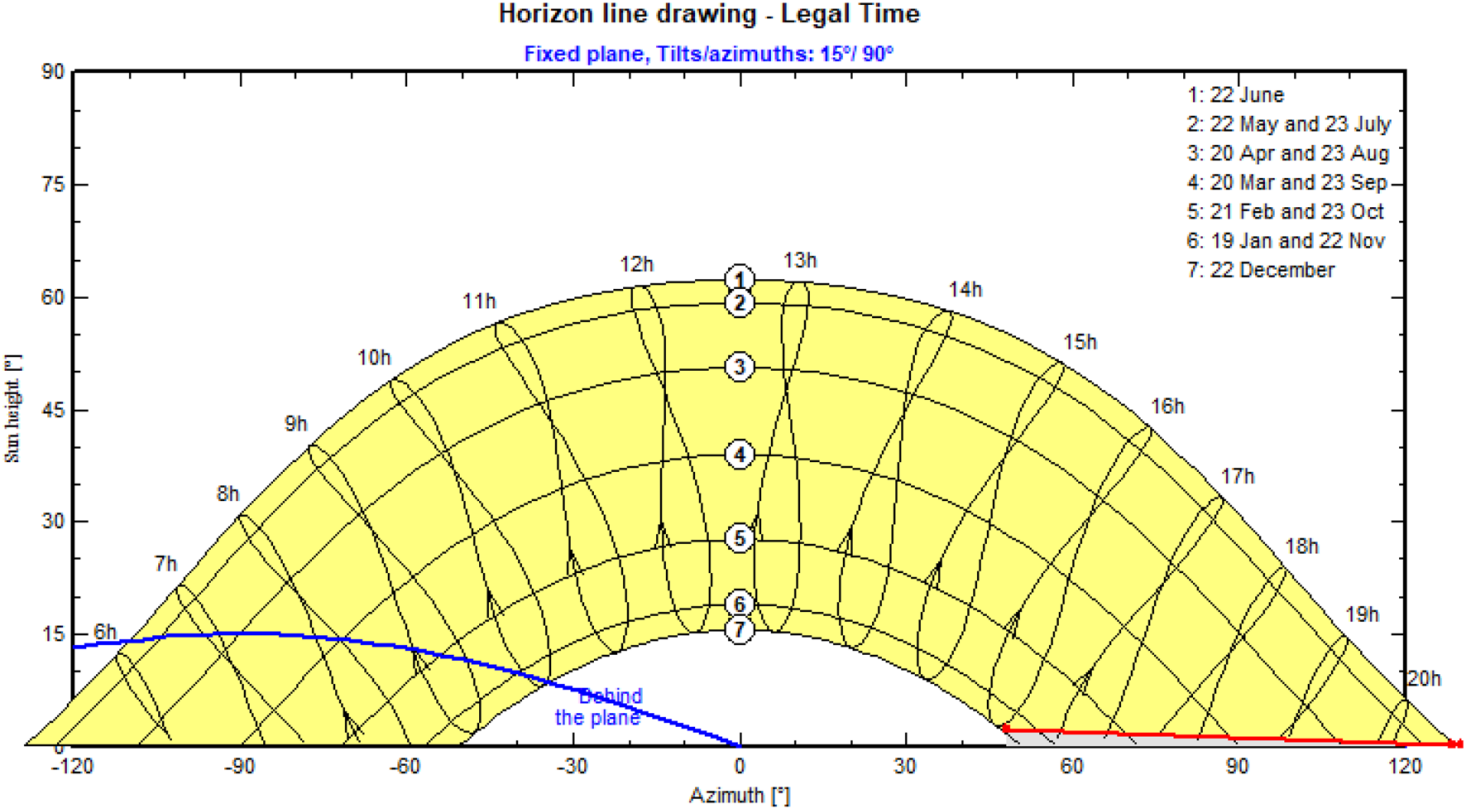
Horizon line shading, based on the simulations conducted in PVsyt.

Simulating large-scale PV systems is challenging since PVsyst can only compute 8 distinct solar systems within the same simulation model. the models with minimal shading explored in SketchUp, are then applied in different settings, such as region, azimuth, tilt, and albedo, using PVsyst. A line horizon shading cut-off is applied to replicate the shade caused by the horizon, using the horizon shading function in PVsyst. In this study, the sun is assumed dispersed from 6 a.m. to 8:30 p.m. in the summer and 8 a.m. to 4:30 p.m. in the winter, if not behind the horizon plane. The horizon line, for Calgary, is drawn with the assumptions indicated in Fig. 4.

For this study, monocrystalline, which are displayed in rooftop and east–west mounts, and bifacial panels, paired with ground mounts on flat rooftops, as well as solar carports and building integrated photovoltaic (BIPV) systems, and solar trees, will be employed to fulfill energy production requirements, depending on the energy demand and qualities of the surface to be placed. Table 5 shows the pros and cons of each selected solar strategy used in the study that would be further useful in the decision-making process for urban planners.

**Table 5:** Neighborhood level solar strategies pros and cons for decision making.

Strategy	Description	Pros	Cons
Monocrystalline			
Tilted rooftops	Installation of solar panels on top of tilted rooftops to capture sufficient solar energy for effective system operation and energy production	Cheap cost of installation, good performance	Might suffer shading from surrounding rooftops and other environmental elements
East–west mount	Installation of solar panel rows on with lower tilting facing east–west	Higher percentage of area usage in flat rooftops, easy access of the modules for maintenance	Lower energy generation performance due to the orientation of the solar panels
Bifacial			
Flat rooftops/ground mount	Installation of solar panel rows in a ground mount using ballasts on top of flat roofs	Higher energy generation performance, lower mutual shading	Lower percentage of area usage in flat rooftops
Solar carport	Installation of solar panels on parking shelters	Use of the parking area as a source of renewable energy generation	Expensive structure, difficult installation, and connection to the grid (usually requires trenching through concrete)
BIPV	Installation of solar panels as elements of buildings	Use of building elements to install solar panels and produce extra energy	Needs custom mounting, usually having a lower generation performance
Solar trees	Installation of solar panels in mounting structure resembling a tree	Good alternative for solar application in green areas, aesthetical appealing	Custom mounting, solar system usually far from grid connection

An albedo of 0.15 is considered for solar carports, which have an asphalt reflecting surface, 0.25 for solar trees (considering grass as the reflecting surface), and 0.9 for flat rooftops assumed to be painted white^38^. For the solar generation simulation, twelve baseline systems are simulated to make solar simulation easier and to create a standard system that can be replicated and adapted to varied urban layouts and applied to each neighborhood according to its suitability. The assumptions used in these baselines are shown in Table 6, considering the region where each neighborhood is located as well.

**Table 6:** PVsyst simulation baseline assumptions^38^.

Simulation S. No	Azimuth (^°^)	Tilt (^°^)	PV Tech	Albedo
Simulation 1	0	25	Monocrystalline	–
Simulation 2	45	25	Monocrystalline	–
Simulation 3	90	25	Monocrystalline	–
Simulation 4	0	25	Bifacial	0.15
Simulation 5	45	25	Bifacial	0.15
Simulation 6	90	25	Bifacial	0.15
Simulation 7	0	25	Bifacial	0.25
Simulation 8	45	25	Bifacial	0.25
Simulation 9	90	25	Bifacial	0.25
Simulation 10	0	25	Bifacial	0.90
Simulation 11	45	25	Bifacial	0.90
Simulation 12	90	25	Bifacial	0.90

The yield of each system is used as a foundation to estimate the energy output of each system. This system configuration is simulated in three distinct azimuths: 0°, 45°, and 90° angles, as well as a 25° plane tilt. A ground cover ratio of 57.1% is chosen for systems put on a level area. This ratio allows a 3.5 m space between modules of 2 m in length, reducing the mutual shade between the modules. The power output of all solar panels is considered as 420 W, with an efficiency of 20.4%. The nominal power per inverter ratio is 1.16, having no overload loss. The reason behind overloading is standard in solar systems design since it allows the inverter to harvest more solar energy during most parts of the day, compensating for the loss due to the inverter clipping when it peaks the energy production output^39^.

### Solar strategies implementation rationale

To evaluate the benefits and drawbacks of each solar strategy when applied in various situations, this study provides a decision-making process that relies on Table 7, in which green signifies a favorable outcome, yellow is an average outcome, and red is a negative outcome.

**Table 7:**
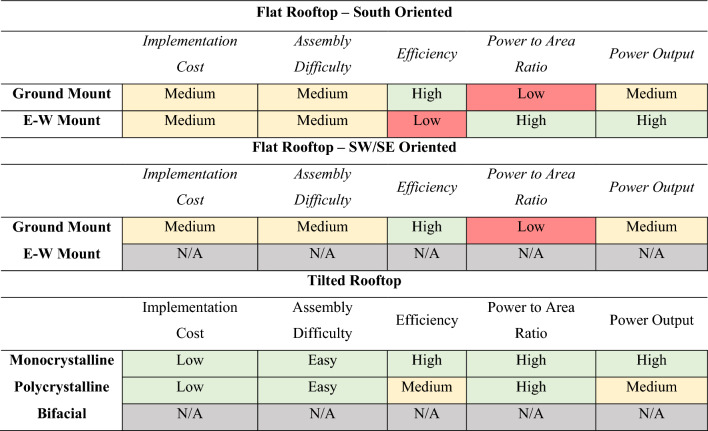
Solar strategy application decision making matrix.

The outcome is assessed based on comparative performance among the various strategies. Table 7 summarizes the decision-making process considering various factors. The criteria employed in evaluating these strategies, namely implementation cost, assembly difficulty, efficiency, power-to-area ratio and power output are briefly explained below.

#### Strategies implementation cost

The decision-making process for implementing solar strategies considers multiple factors. The cost of implementing such technology is the first one to be analyzed. Considering tilted rooftops, the cost of installing solar panels is low: $1.00 to $1.50 per watt for monocrystalline modules, $0.90 to $1.00 per watt for polycrystalline, and $2.41 to $3.42 per watt for bifacial solar panels^40, 41^. Since the structures to install solar systems in tilted rooftops are cheap, monocrystalline and polycrystalline are rated as “low” in the cost category, while bifacial is rated as “medium”.

When considering the installation of solar systems in flat roofs, the cost may vary depending on the design due to the elevated ground mount structures needed to space and tilt the modules. Assuming that there is an extra cost for such structures, systems that require a ground mount are also rated “medium” in the cost factor.

Other than flat and tilted rooftops, there are options to implement solar systems, such as solar carports in parking lots, solar trees, and BIPV using solar panels as building elements. All the strategies cited above require custom designs and structure manufacturing, as well as specialized labor to install, and thus are rated as “high” in the cost factor of the implementation rationale.

#### Assembly difficulty

Following the same approach as the cost factor, assembly is considered when choosing the best solar strategy for each situation. Installing solar panels on tilted rooftops is the most common type of installation, having standard clamps and an anchoring system that makes the process very fast and easy, therefore is rated as “easy”. Ground mounts require assembly of the ground structures, anchoring into the slab, and waterproofing it. The installation process requires more material and more attention in the assembly, consequently being rated as “medium”.

Again, custom designs like carports, solar trees, and BIPV takes usually more time to install, assemble and design due to the complexity of the structures to install. Also, most of it requires specialized labor. Consequently, this type of strategy is rated as “hard”.

#### Efficiency

On tilted rooftops, monocrystalline has a better performance due to the pureness of the silicon used in the manufacturing process, therefore is rated as “high”^42^. Polycrystalline has a lower efficiency than monocrystalline, rated as “medium”. Bifacial modules are not considered in tilted rooftops because their rear surface does not face a reflecting surface, so it makes no use of their advantage over similar technologies.

On flat surfaces, ground-mounted systems have a better performance than east–west mount because of the orientation of the arrays and optimized tilting, ranked as “high”. East and west mount have poor orientation, therefore having a “low” efficiency.

Since custom designs such as carports, solar trees, and BIPV are sometimes the only option to be implemented in some environments, it is assumed that there is the freedom to choose whatever technology fits best under the determined circumstance, despite the efficiency of the systems. Solar carports efficiency is usually “high” but might be affected if there are nearby buildings shading the parking area. BIPV performance depends heavily on the method of implementation including the tilt angle of the surface used for the integration of this PV system, therefore each BIPV system proposed in the study will be evaluated and simulated separately and not included in this solar strategy implementation rationale. Solar trees are assumed “high” since they can be placed in no shading areas, but the albedo from grass is low.

#### Power to area ratio

**Figure 5:**
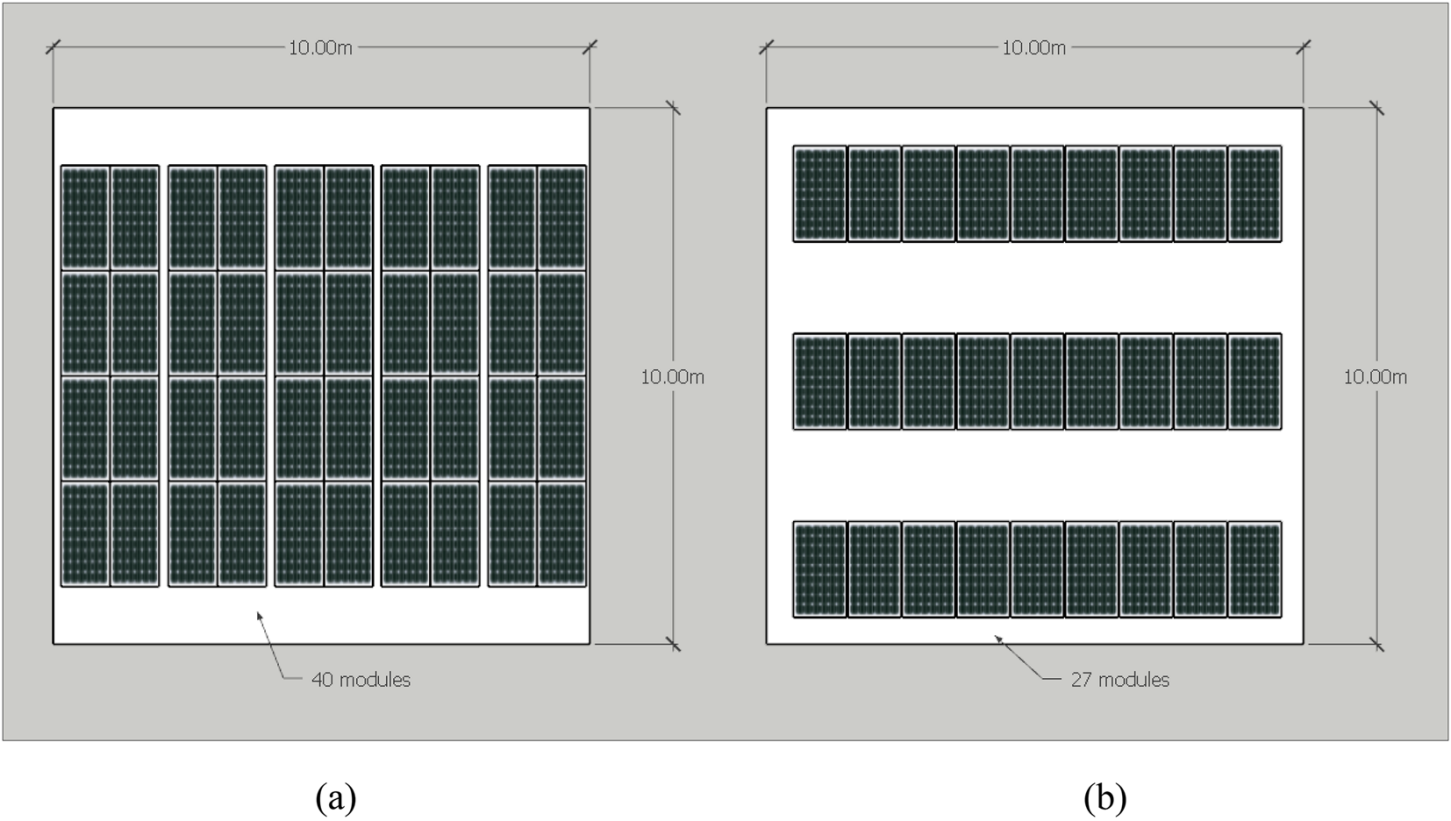
Power to area ratio comparison: (**a**) east–west mount and (**b**) ground mount on the right.

This category factors in the amount of solar power that can be installed in each area. Since all the modules are the same size, tilted rooftops are not applicable to this category. Ground mounts used in flat rooftops have better efficiency, however, it needs more spacing due to mutual shading, having a lower power per area ratio than east–west mount. Figure 5 illustrates how many solar panels are suitable in a 100 m^2^ area. After the comparison, the east–west mount is rated as “high”, while the ground mount is rated as “low”.

#### Power output

The efficiency and power output of each technology is simulated and compared using PVSyst. Thus, the power-to-area ratio is determined by designing each strategy for the rooftops available in each neighborhood using SketchUp as a modeling tool. After simulating all the technologies in different environments, the following conclusions are achieved: for tilted rooftops, monocrystalline is the technology that has the better energy generation, therefore rated as “high”. For flat rooftops, the east–west mount has a slightly better power output, even though its efficiency is lower.

When the flat rooftop is not oriented south, ground mount power output is better than east–west mount. Also, this study does not consider systems that are oriented north, northeast, and northwest, so an east–west mount is not used on flat rooftops that are not south-oriented because half of the system would not be in an acceptable orientation. Solar carports, solar trees, and BIPV are used as suitable disregarding the efficiency or power output of the system since it is considered as an alternative system to be employed in alternate locations.

Moreover, East–West mount PV employs monocrystalline modules due to the low lighting incidence of its back surface, whereas ground mount PV uses bifacial due to the considerable exposure to light reflection from its surface. Flat south-facing rooftops will benefit more from East–West mount PV since the power output and area ratio is higher than Ground Mount PV by being the same effect on cost and installation. When the building is not south-faced, east–west mounts have a large loss in power production and therefore do not meet with the premise of this study of not having solar systems facing northwest or northeast. When analyzing the available rooftop systems, monocrystalline came out ahead because it presents the biggest benefits when compared to similar technologies.

## Results and discussion

The current paper focuses on the energy modeling of existing Canadian neighborhoods in various climatic zones through detailed 3D energy models accounting for real neighborhood geometries, envelope characteristics, mutual shading, occupancies, energy use schedules, etc. Thereafter, the impact of building retrofitting and solar strategies (using decision-making matrix) is evaluated in achieving solar neighborhoods and net-zero energy. This section presents the key results of this work.

### Energy models validation

This section presents the results of the base cases of the specific neighborhoods and the improved neighborhoods (presenting improved building envelopes) and compares their energy performance to the baselines developed using the NRCan database.

The following simulation results display the amount of energy consumed by different types of buildings detached houses (DH), and attached houses, (AH) in their typical and retrofitted form and compare them to the NRCan baseline, explained in Sect. “Model validation”. This comparison allows an understanding of how each of the studied urban designs performs when compared to the average energy performance of each province. Moreover, it shows the impact of the implementation of an improved building envelope on the neighborhood performance representing various urban designs and regions. The larger the energy consumption of the neighborhood, the more critical the implementation of a PV system to achieve net-zero energy.

**Figure 6:**
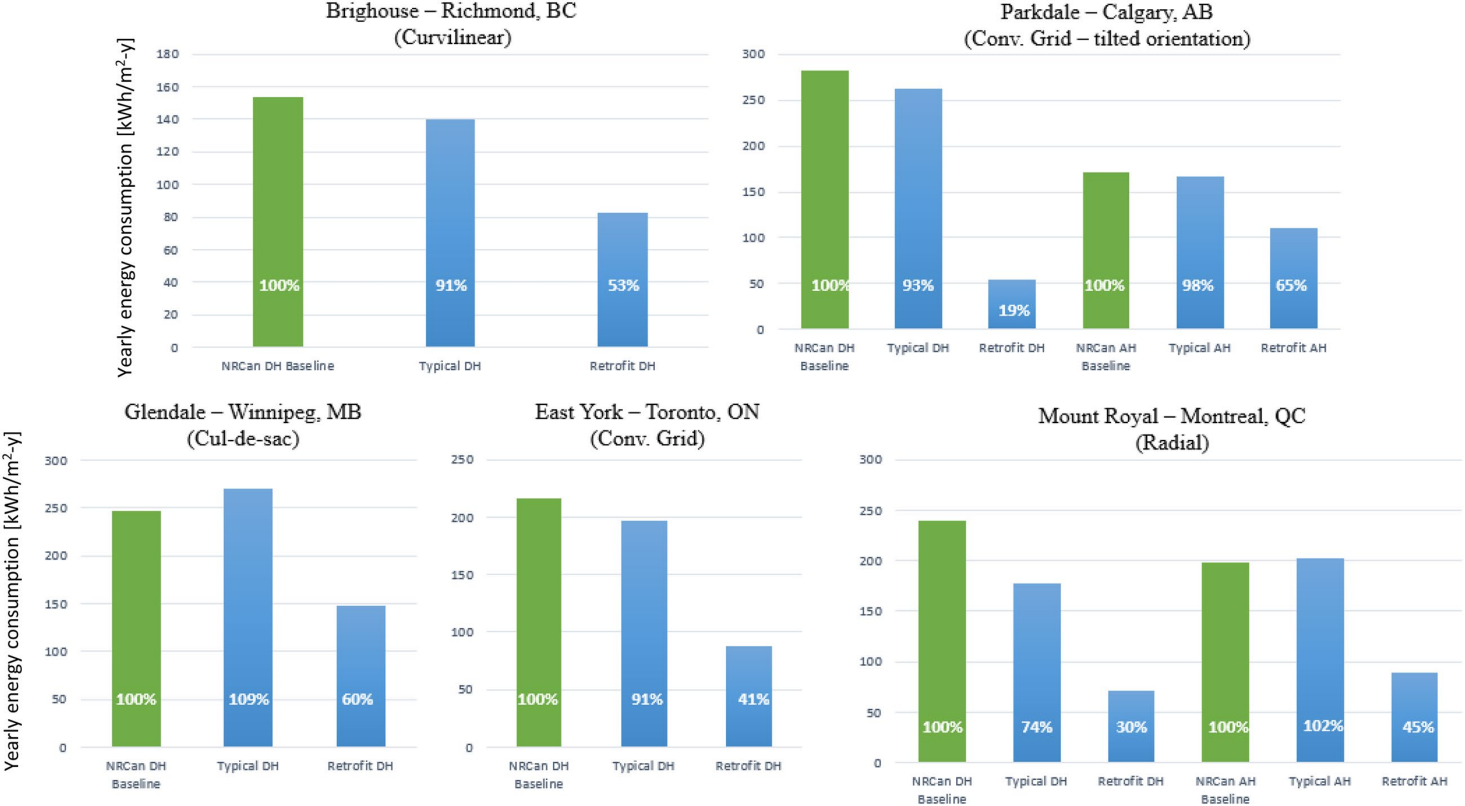
Yearly energy consumption comparison of various neighborhoods [kWh/m^2^/year].

The results illustrated in Fig. 6 show that the energy consumption of neighborhoods employing typical constructions conforms to the performance of the baseline (within a 10% difference). This is except for Mount Royal detached houses, which are 26% more efficient that the baseline model. This performance is expected due to the larger size of the building (the average detached house in Mount Royal is ~ 250% larger, compared to the average in Quebec). Since the interior divisions of the homes are not considered in the simulation, the heating system has a better performance in one big zone instead of multiple sections of a house, therefore having a better energy performance even though the comparison is done using kWh/m^2^. All models use the same assumptions and settings. The percentage reduction in energy consumption after applying the improved building envelope varies among the different locations and building designs. For instance, the energy consumption of detached houses is reduced by about 60%, while attached houses have an average energy consumption reduction of 45%.

### Solar strategies application and energy performance

**Figure 7:**
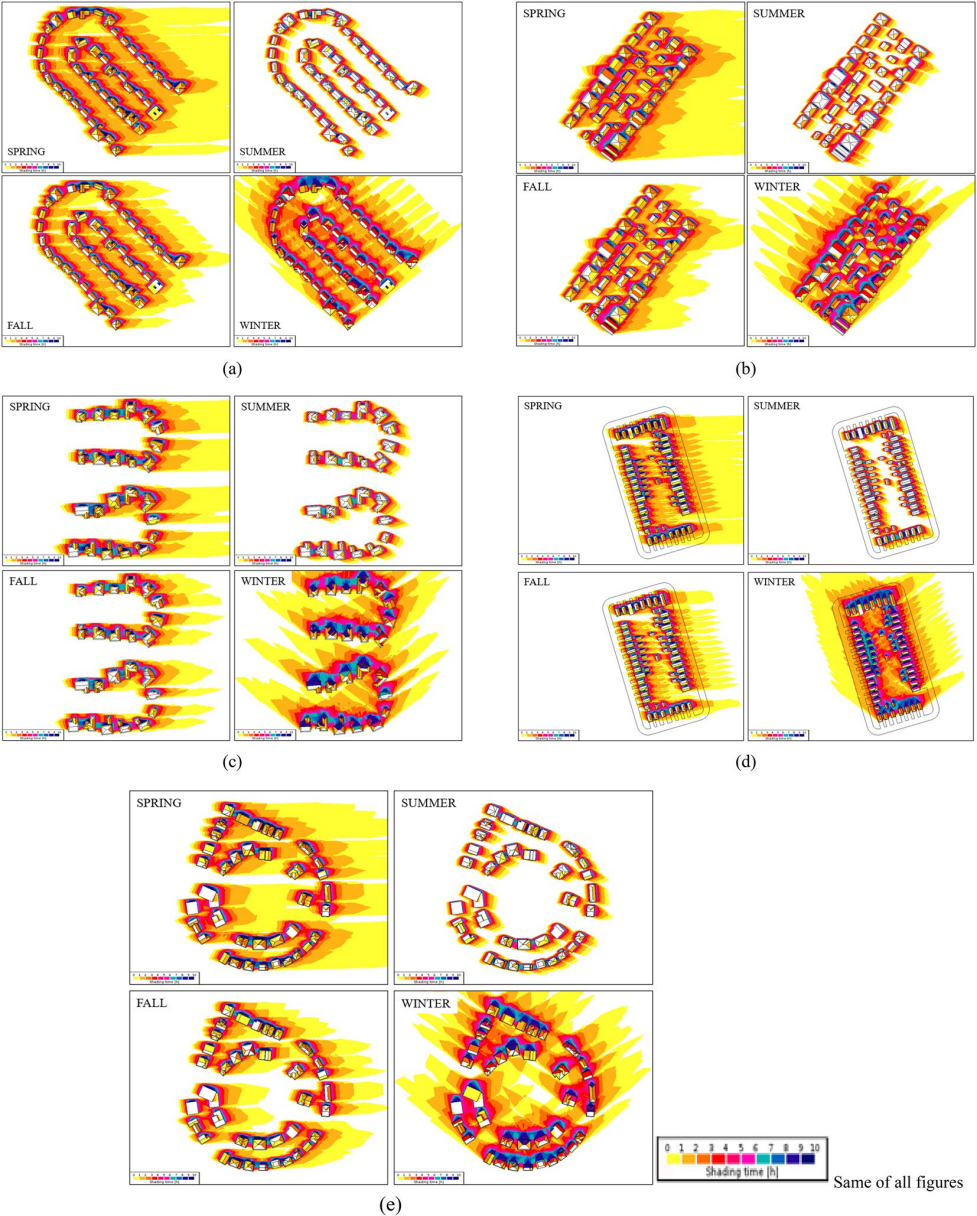
Example of neighborhood scale shading analysis.

This section describes the impact of various solar strategies on onsite energy generation for various considered neighborhoods. As mentioned in the previous section, a shading analysis is carried out to help decision making about solar strategies. Figure 7 shows an example of the shading analysis of one of the studied neighborhoods—Parkdale. In the figure it is evident to identify areas of the deployment of solar strategies based on solar access. Further, the results of solar strategies application are presented neighborhood-wise in this section.

#### Brighouse–Richmond, BC (curvilinear loop)

**Figure 8:**
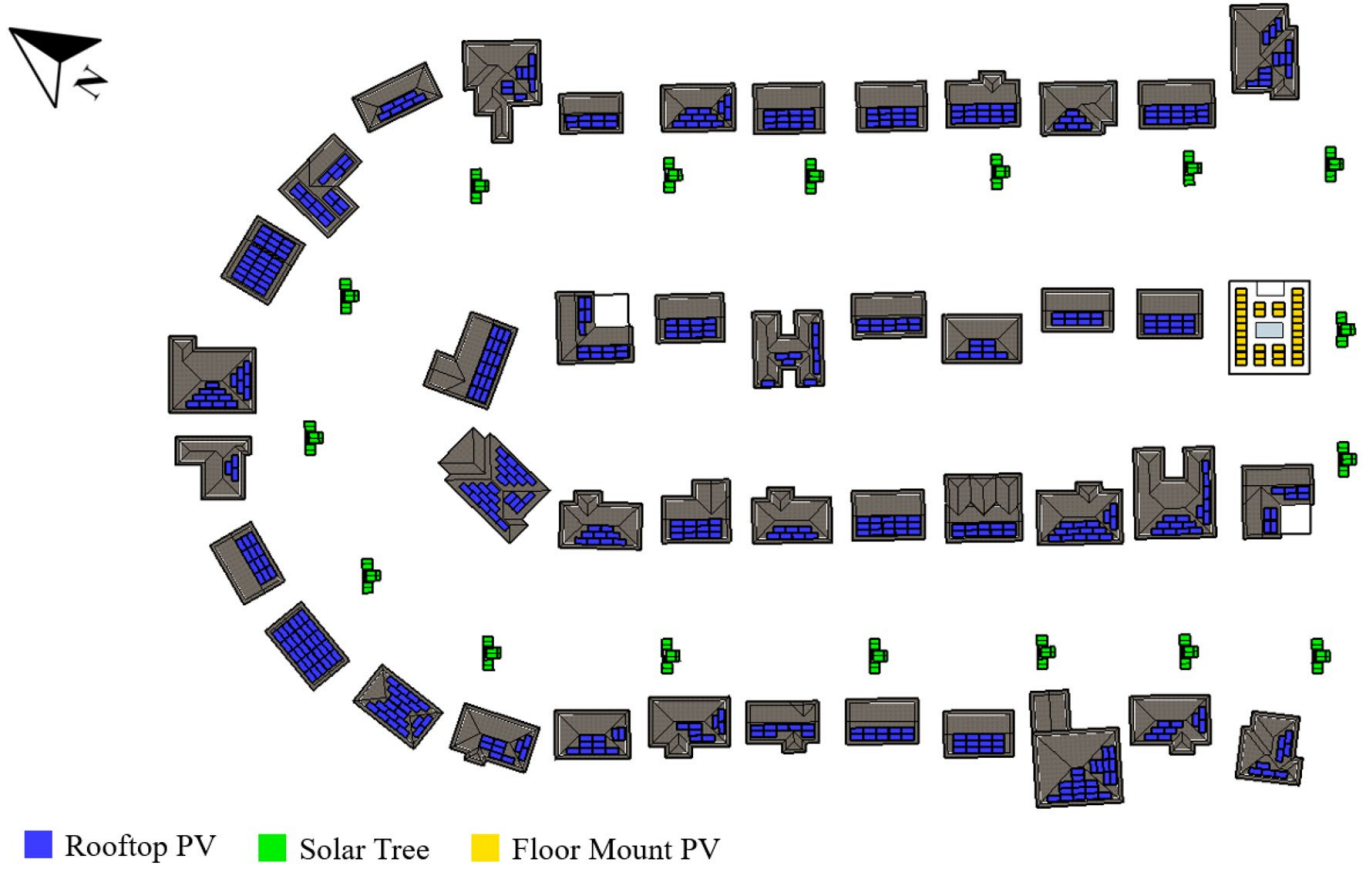
Brighouse (curvilinear loop street layout) neighborhood proposed solar strategies.

The first analyzed is the Brighouse neighborhood with a curvilinear loop street layout, located in Richmond, BC. As shown in Fig. 8, the proposed solar systems are monocrystalline roofs with a total capacity of 224.7 kWp, with 12.6 kWp facing south, 161.7 kWp tilted between 30° and 60°, and 50.4 kWp inclined between 60° and 90°. Bifacial modules for flat roofs mounted on the ground have a slanted orientation and a power output of 13 kWp. Finally, 17 solar trees totaling 57.1 kWp are planned for the streets. Solar panels comprise 295.3 kWp and generate 283,970 kWh per year.

#### Parkdale–Calgary, AB (conventional grid–titled orientation)

**Figure 9:**
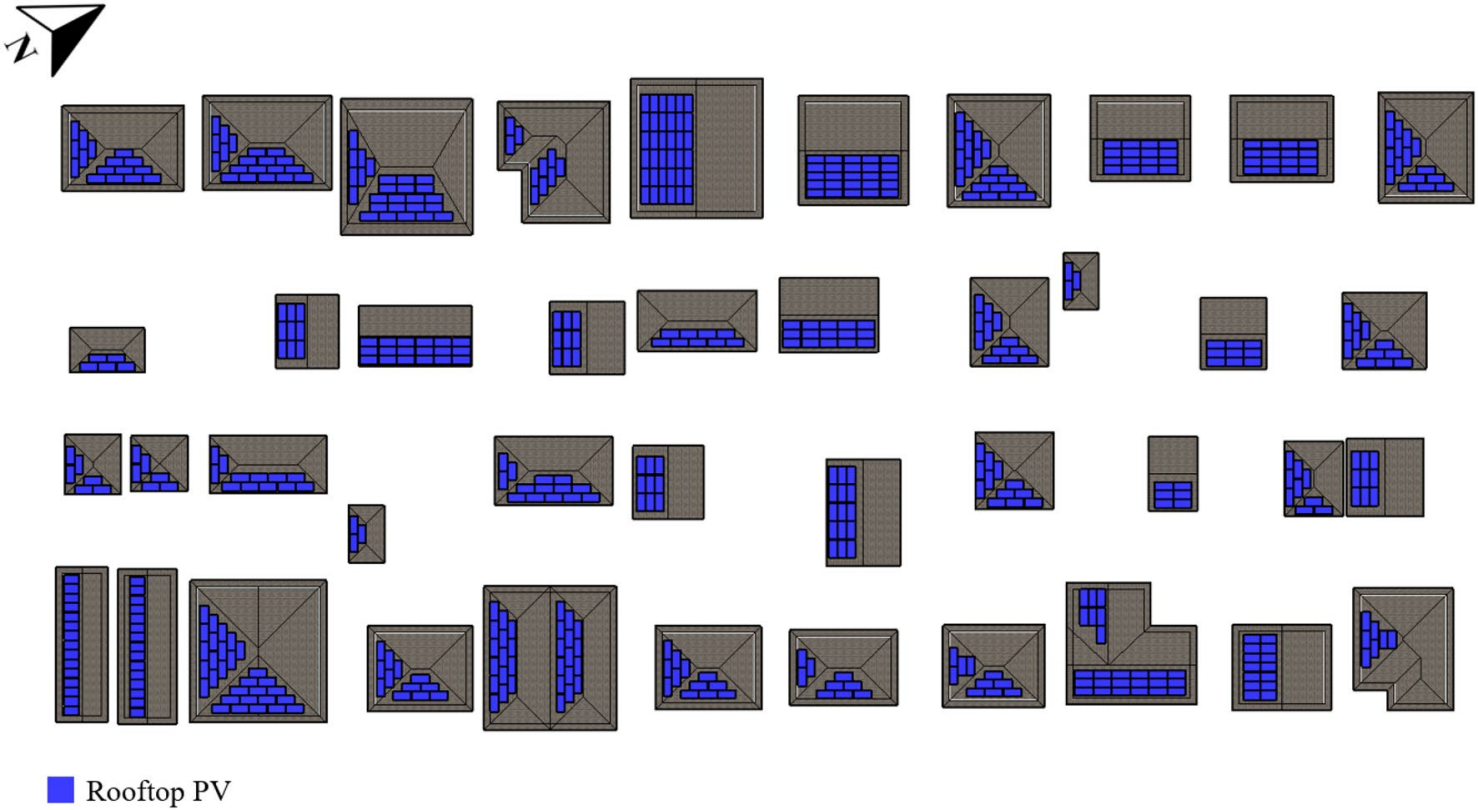
Parkdale (conventional grid with tilted orientation street layout) neighborhood proposed solar strategies.

The residential urban arrangement of Parkdale, located in Calgary, AB, and with a conventional grid with a tilted orientation street layout, allows only the use of PV modules on rooftops, as well as roofs from detached garages in the back alley, as indicated in the system display in Fig. 9. Solar power is installed on the rooftops in the amount of 244.4 kWp. All solar systems in the area are oriented in the same direction since the neighborhood is inclined between 45°. The total energy production is 267,173 kWh per year.

#### Glendale–Winnipeg, MB (cul-de-sac)

**Figure 10:**
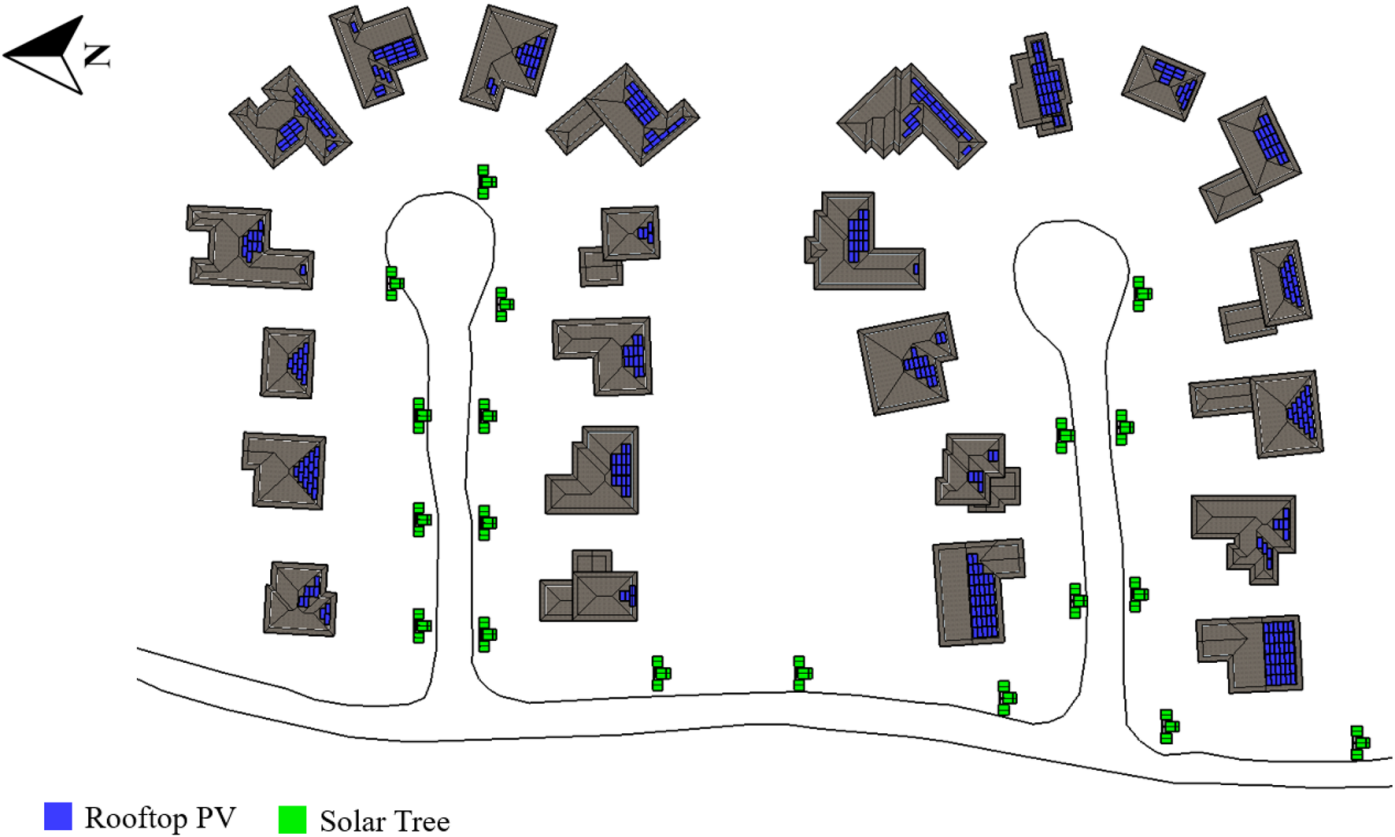
Glendale (cul-de-sac street layout) neighborhood proposed solar strategies.

The Glendale neighborhood is located in Winnipeg, MB, and it has a cul-de-sac street layout. Monocrystalline modules on the rooftops, totaling 160.0 kWp, are proposed, with 120.54 kWp facing south and 39.5 kWp inclined between 30° and 60°. Additionally, 19 solar trees totaling 63.84 kWp are planned for the streets. The distribution of systems in the neighborhood is illustrated in Fig. 10. The entire installed solar power is 223.9 kWp, which generates 271,809 kWh per year. As per PV potential and solar resources map by Natural Resources Canada, Winnipeg falls under high potential region, hence the energy generation from solar installations is around 1.2 MWh/kWp.

#### East York–Toronto, ON (conventional grid)

**Figure 11:**
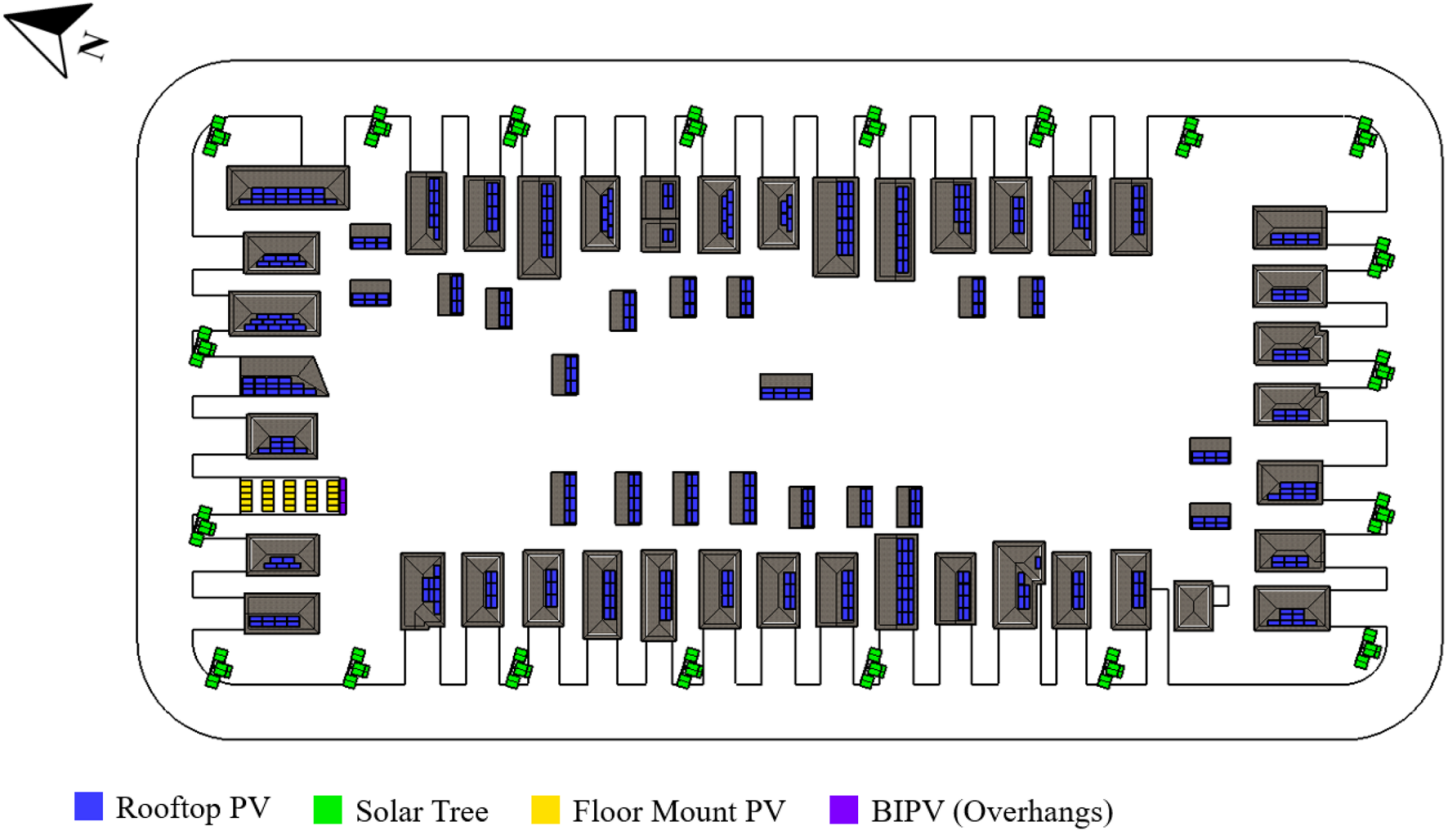
East York (conventional grid street layout) neighborhood proposed solar strategies.

The East York neighborhood is in Toronto, ON, and has a conventional grid street layout (Fig. 11). Apart from one building with a flat roof, the urban layout of East York only allowed proposed PV modules on rooftops, thus ground mount systems and BIPV, as well as some roofs from detached garages in the back alley, were viable. On the roofs, monocrystalline 420 W modules are used to generate 200.3 kWp, with 134.4 kWp orientated south and the remainder positioned between 60° and 90°. There's also a 10.5 kWp bifacial ground mount system and a 2.5 kWp BIPV system that acts as overhangs facing south. Finally, 20 solar trees are installed along the sidewalk, with a total solar power of 67.2 kWp. All solar panels have total capacity of 280.56 kWp and generate 296,930 kWh per year.

#### Mount Royal–Montreal, QC (radial)

**Figure 12:**
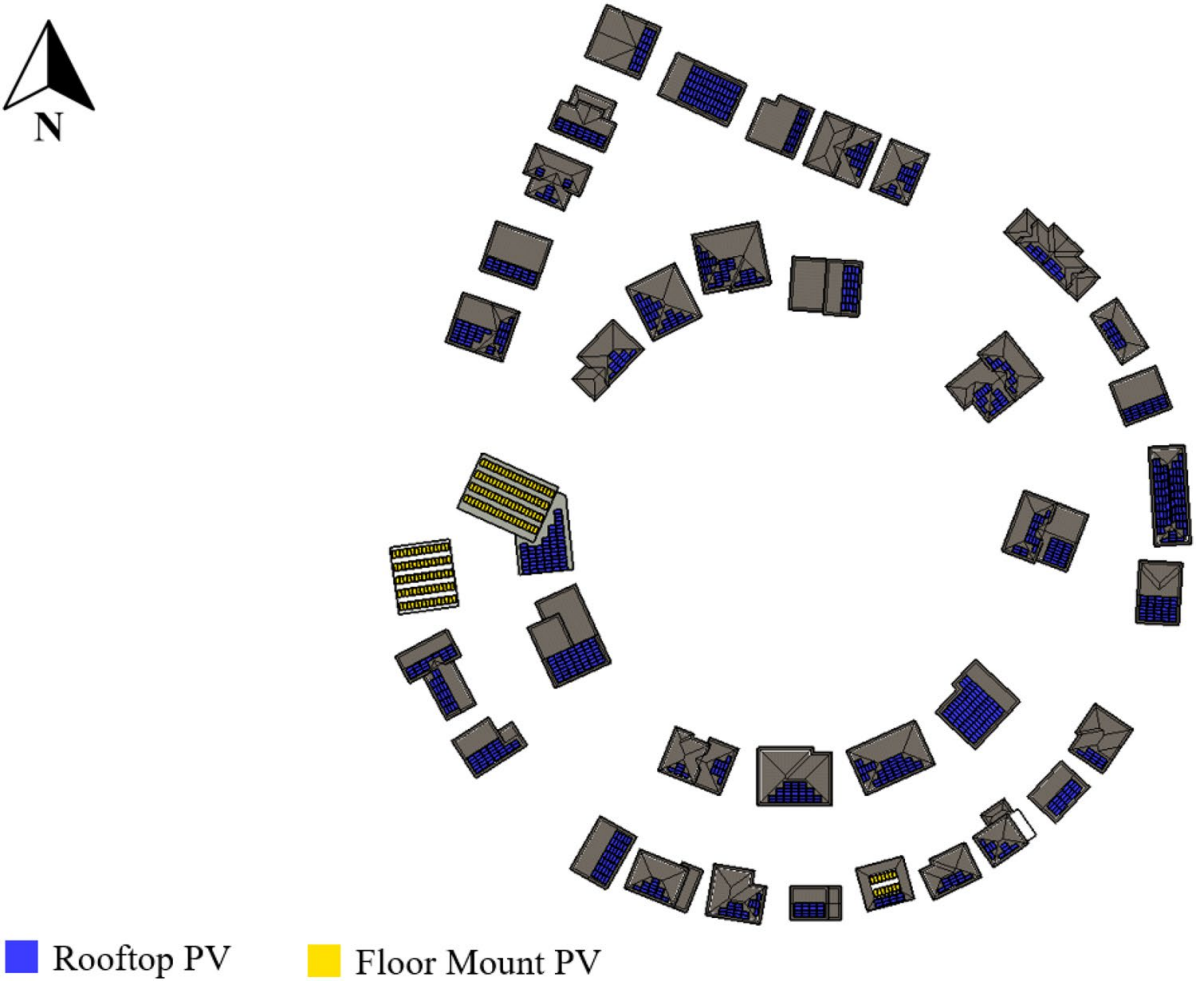
Mount Royal (radial grid street layout) neighborhood proposed solar strategies.

For the Mount Royal neighborhood, located in Montreal, QC, with a radial street layout, the solar strategies proposed are monocrystalline rooftops systems with a total capacity of 436.4 kWp, including 183.54 kWp facing south, 160.4 kWp tilted between 30° and 60°, and 92.4 kWp inclined between 60° and 90°. Bifacial ground-mount modules for flat roofs have a tilted orientation as well, with a total power of 73.0 kWp, with 37.1 kWp optimally orientated south and 35 kWp oriented between 30° and 60°. The distribution of systems in the neighborhood is depicted in Fig. 12. Solar power of 509.0 kWp has been installed, yielding 549,347 kWh per year.

### Comparison of various neighborhood layouts

This section aims to discuss the key findings of the results presented above to point out the differences that each urban layout has on energy performance and solar potential, as well as to assess the potential and limitations of this work.

#### Solar potential

**Figure 13:**
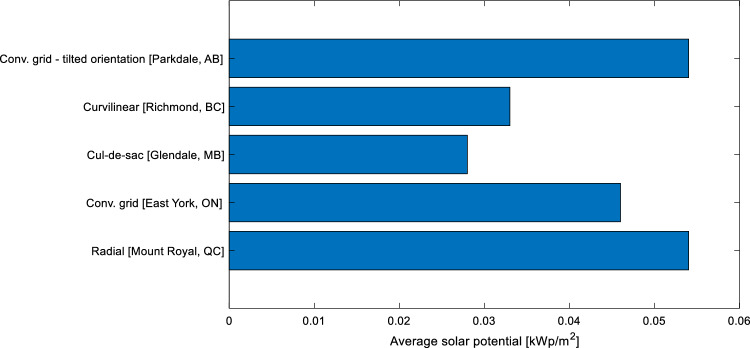
Energy performance for various neighborhoods under various scenarios.

Figure 13 presents the comparison among the neighborhoods. The urban layout with the most solar potential in this study is the conventional grid with tilted orientation (Parkdale) and the radial (Mount Royal), both with an installed capacity of 0.054 kWp/m^2^. Conventional grid (East York) also presented a high capacity with 0.046 kWp/m^2^, while the curvilinear loop (Richmond) and cul-de-sac (Glendale) had the worst average solar potential, with 0.033 and 0.028 kWp/m^2^, respectively.

#### Energy demand reduction

**Figure 14:**
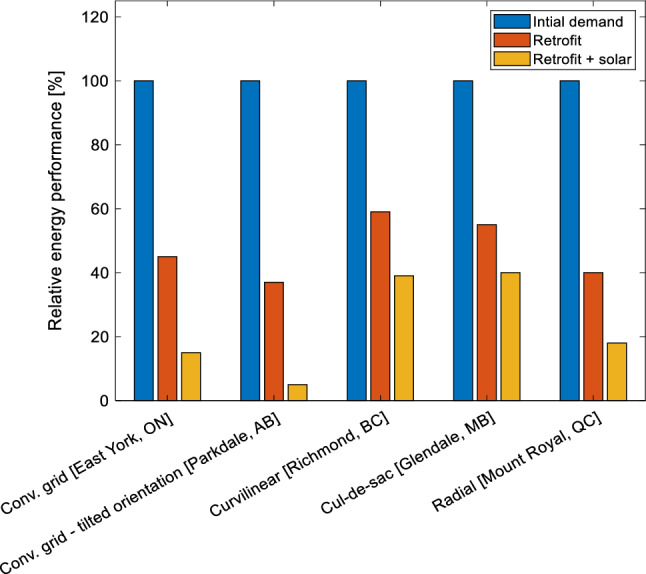
Average solar potential comparison of various neighborhoods having different street layouts [kWp/m^2^].

Figure 14 illustrates the amount of reduction in the energy demand, achieved at each stage of the study. The overall energy demand reduction varies by up to 35% among the studied neighborhoods. The neighborhood that had the most energy reduction is Parkdale (Conventional Grid with tilted orientation), with 95% of its original demand, while the one with the least reduction in Glendale (Cul-de-Sac). The retrofit of the building’s envelope is more effective for Parkdale (Conventional Grid with tilted orientation) and East York (Conventional Grid), achieving a 63% and 55% reduction as compared to the original demand, respectively. The least energy reduction of 41% is associated with Brighouse (Curvilinear Street layout).

Considering the implementation of solar strategies, the neighborhood with the best performance is Parkdale and East York, with a reduction of 32% and 30% of the net energy consumption. The neighborhood with a less favourable solar installation area (0.028 kWp/m^2^) is Glendale (cul-de-sac), achieving 15% less net energy consumption upon retrofitting and solar installations in comparison with only retrofitting case.

#### Comparison to existing data

Comparing the results to the baseline (model built employing the database created by Natural Resources Canada for energy use statistics^30^, a good fit is generally observed with a maximum difference of 10%. This is observed for all the neighborhoods, except for Mount Royal detached houses, which is 26% more efficient that the baseline model. This inconsistency is due to the larger size of the building (the average detached house in Mount Royal is ~ 250% larger, compared to the average in Quebec).

### Design, implementation, and limitations

In this sub-section, various aspects of the study are discussed highlighting addressed literature gaps and usefulness for professionals.

**Figure 15:**
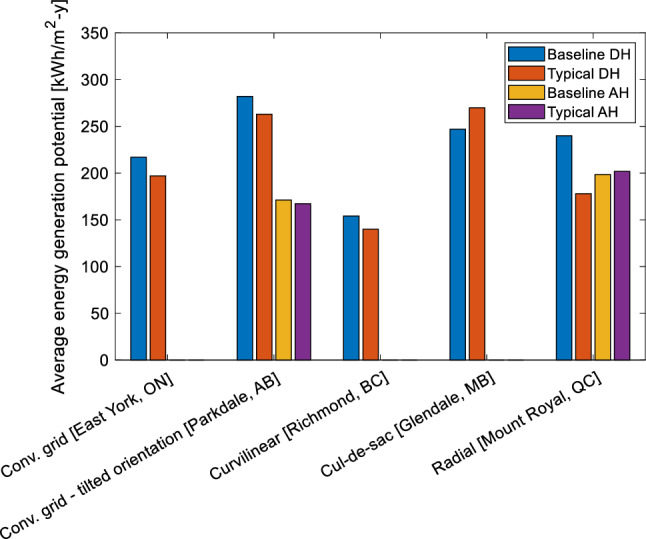
Energy performance comparison of various neighborhoods having different layouts [kWh/m^2^/year].

- Comparing the energy performance of all the urban layouts, some conclusions can be reached. Conventional grid with tilted orientation, conventional grid and curvilinear have a slightly better performance as compared to the baseline, while the radial layout presents the best performance among the neighborhoods. The cul-de-sac layout is the only urban form that has an initial energy performance worst than the baseline, and worst performance as compared to the other neighborhoods, both in solar potential and energy performance. The comparison results are shown in Fig. 15. For comparison terms, the unit used is kWh/m^2^/y, since the average floor area of each building is different than the provincial average floor area, used to model the baseline. These insights will be useful for the urban planners in planning new neighborhood developments.
- In this study, most commonly applied neighborhood patterns are identified and analysed. Addressing the gap in literature, a systematic methodological framework is provided in this work from neighborhood data collection (using proposed matrix) to energy modeling and simulation. This generalized framework can be easily adopted by urban planners and energy modelers to assess the impact of retrofitting and solar strategies to achieve net-zero energy status for any residential neighborhood. These however are restricted in this work to residential neighborhoods. Potentially the methodology can be extended to other types of neighborhoods such as mixed-use, commercial neighborhoods, high density business districts etc.
- The study proposes decision making framework for solar strategies applications in the presented archetypes that is rarely available in the literature. A simple decision-making tool is proposed to assist in identifying the most suitable and beneficial strategies for each of those archetypes, in order to achieve the objective of net-zero energy. This decision-making tool can be further developed to allow weighting various options and to determine the most optimal solutions according to various objectives, and according to different criteria such as ease of implementation, accessibility, and cost. In addition, other solar strategies can be explored, including advanced passive strategies to understand their performance in various layouts, and their potential application and viability in existing neighborhoods.
- The main limitation of this work is related to reliable data on the energy performance of the existing building stock. The existing information is mostly related to specific types of residential buildings, while information on commercial buildings is extremely limited. On the other hand, the information on residential buildings is general, as it does not link performance to specific design aspects of building envelope and energy systems. This made benchmarking a challenging task. As more detailed information on different type of buildings become available, the design of other neighborhood archetypes and analyzing them for improved performance becomes more feasible.
- Despite its limitation, this work can give valuable insight to professionals including planners and architects into the impact of neighborhood layout and overall spatial design, as well as building design on energy performance and potential solar energy. This can significantly assist urban planners in developing more guided solutions toward achieving cities with reduced carbon footprint.

## Conclusion

This study investigates the performance of selected neighborhoods, representative of urban layouts in Canada in order to develop solar strategies to attain sustainable communities. The work proposes generalized systematic framework to simultaneously analyze retrofitting and solar strategies using level of detail 3 and multi-domain energy models. A novel data collection matrix for neighborhood energy modeling is also presented in addition to solar strategies decision making framework for the professionals. The methodology is demonstrated on various selected neighborhoods characterized by the conventional grid, conventional grid with a tilted orientation, cul-de-sac, curvilinear loop, and radial. The energy performance of these neighborhoods is simulated in three stages: (a) with typical construction materials employed in the building envelope of the buildings; (b) with improved building envelope incorporating highly insulated materials; and (c) with the implementation of solar strategies to reduce the overall energy demand, aiming to achieve net-zero energy. The results show that the overall energy performance of the conventional grid with tilted orientation, as well as its solar potential, and energy reduction after implementing retrofit strategies, is the best performing as compared to other urban layouts (only 5% of energy demands to be met by external resources after retrofitting and solar strategies). Its symmetrical form is advantageous when designing solar systems and its tilted orientation allows for implementation the large number of solar panels since both southeast and southwest orientation roofs can be used. Conventional grid layout has a good balance between energy efficiency and solar potential, being the second closest to reaching net-zero energy, enabling the implementation of a good mix of different solar strategies due to their linear design. In addition, the radial layout shows promising results, although it has a lower density, which impacts environmental aspects and sustainability metrics. As expected, Cul-de-Sac and Curvilinear are the least performing among all studied neighborhoods. Employing the proposed methodology, additional investigation needs to be carried out to take into account various global archetypes capturing other urban layouts.

### Supplementary Information

Supplementary Information.

## Data Availability

The datasets used and/or analysed during the current study available from the corresponding author on reasonable request.

## Abbreviations

AB: Alberta
AH: Attached house
BC: British Columbia
BIPV: Building integrated photovoltaic
CEEDS: Canadian energy and engineering data sets
DH: Detached house
GHG: Green house gases
GIS: Geographic information system
IEA: International energy agency
LoD: Level of detail
MB: Manitoba
NRCan: Natural resources Canada
OSB: Oriented strand board
PV: Photovoltaic
QC: Quebec
