# Supplementary figures and images for: Solar neighborhoods: the impact of urban layout on a large-scale solar strategies application
Source: Sci Rep. 2023 Nov 1;13:18843. doi: 10.1038/s41598-023-43348-8 (PMC10620396; doi:10.1038/s41598-023-43348-8)

**APPENDIX A**

**
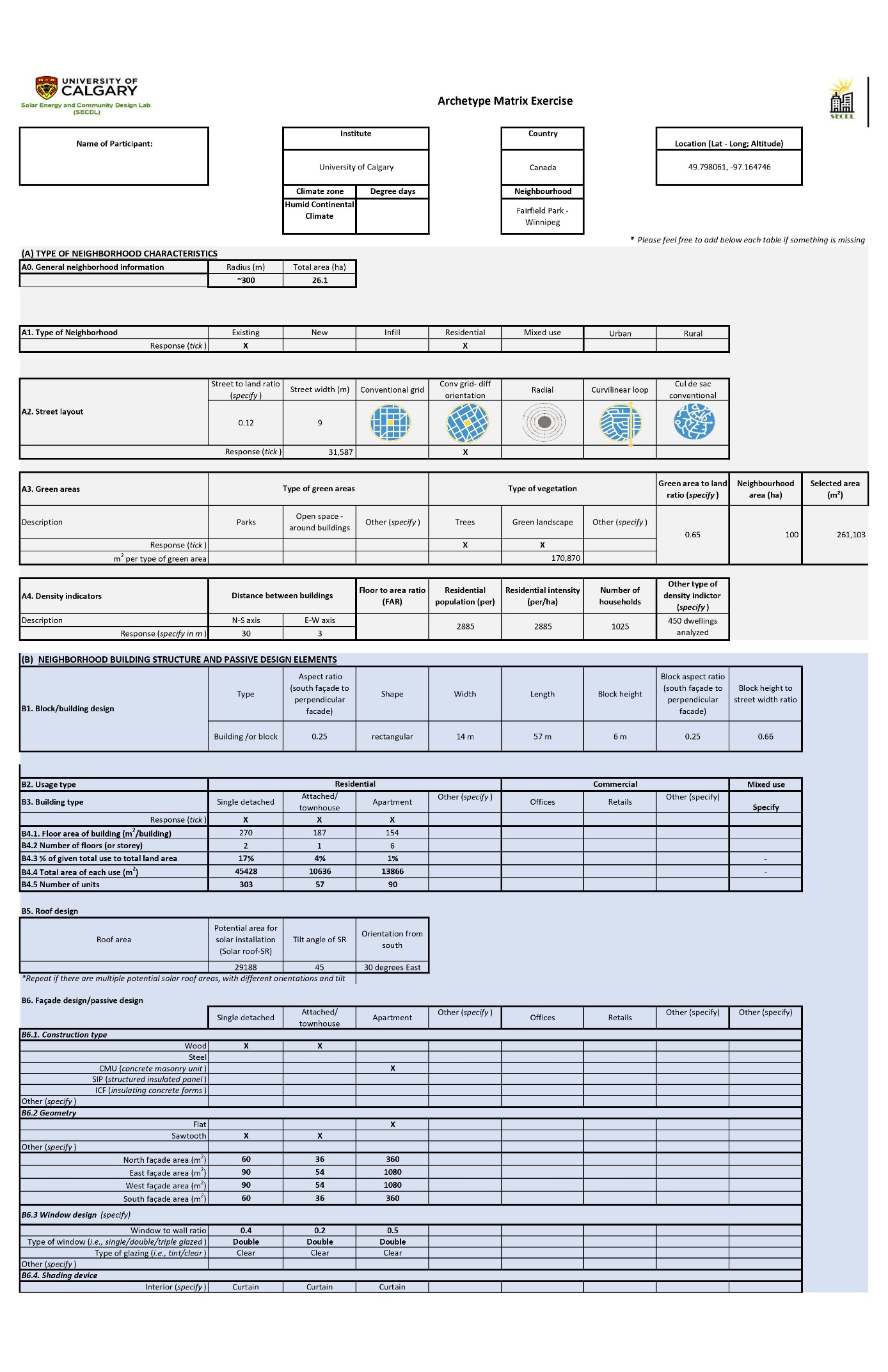
**

**
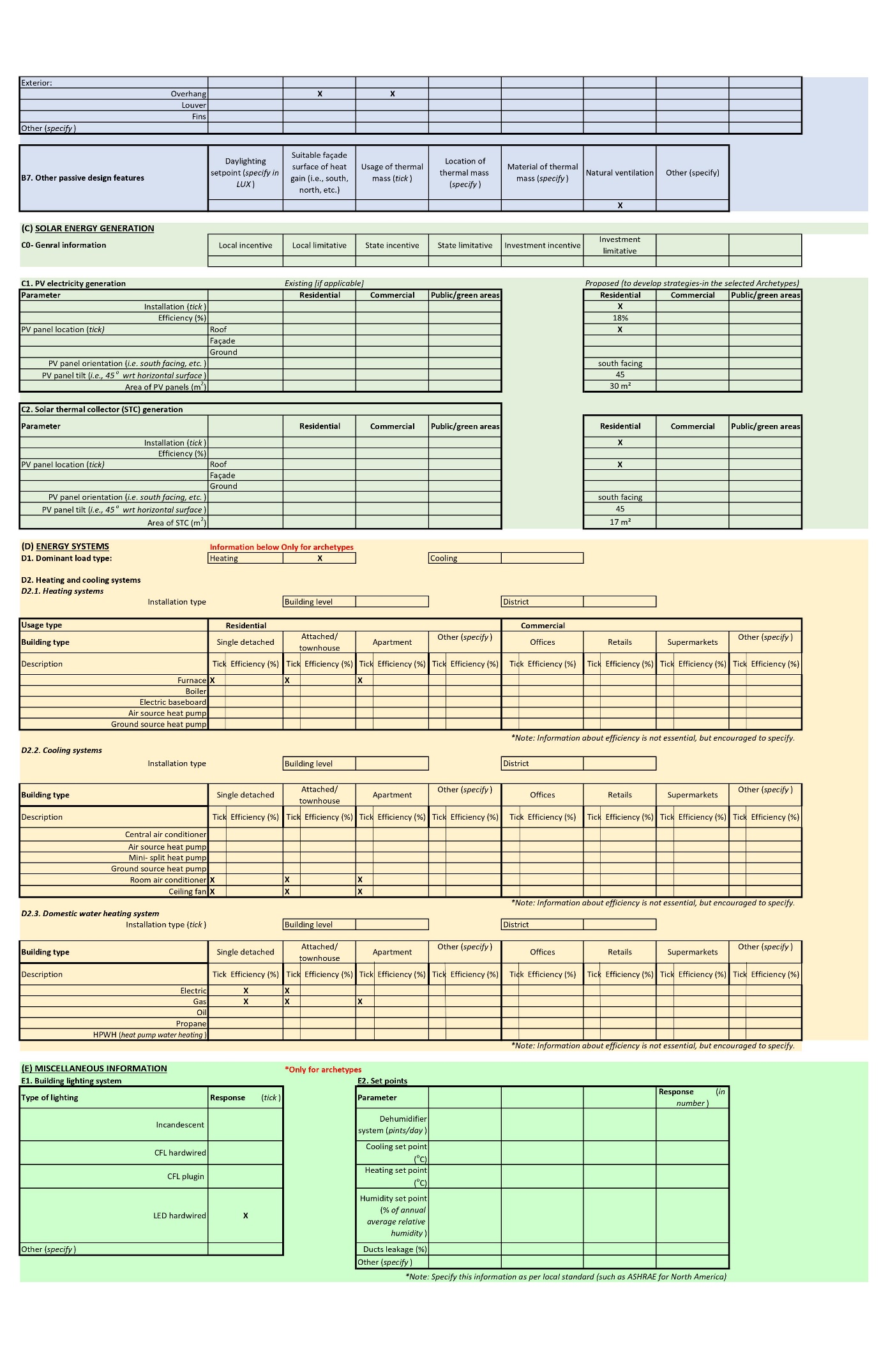
**

**
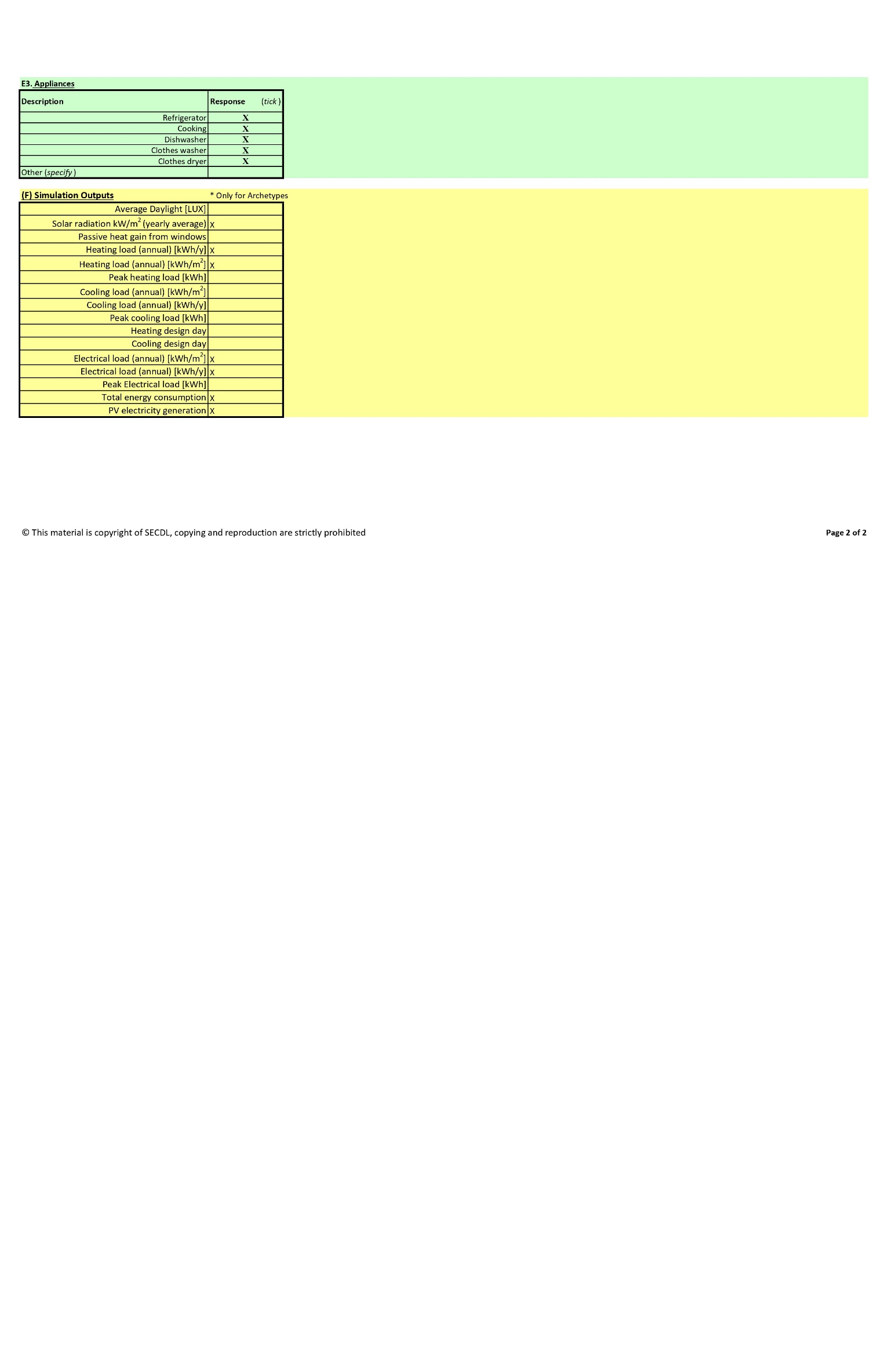
**

Supplement: Supplementary file 1 — Supplementary Information. [file 41598_2023_43348_MOESM1_ESM.docx]
